# A Rare CTBP1-Related Neurodevelopmental Disorder Is Associated with Impaired Mitochondrial Bioenergetics: A Functional Case Report

**DOI:** 10.3390/ijms27094003

**Published:** 2026-04-29

**Authors:** Zdravko Ivanov, Maria Gevezova, Iliyana Pacheva, Kostadin Ketev, Lyubov Chochkova-Bukova, Victoria Sarafian, Ivan Ivanov

**Affiliations:** 1 Pediatrics Clinic, St. George University Hospital, 4002 Plovdiv, Bulgaria; iliyana.pacheva@mu-plovdiv.bg (I.P.); kostadin.ketev@mu-plovdiv.bg (K.K.); lyubov.chochkova@mu-plovdiv.bg (L.C.-B.); 2 Department of Pediatrics, Medical University of Plovdiv, 4002 Plovdiv, Bulgaria; 3 Department of Medical Biology, Medical University of Plovdiv, 4002 Plovdiv, Bulgaria; mariya.gevezova@mu-plovdiv.bg (M.G.); victoria.sarafian@mu-plovdiv.bg (V.S.); 4 Research Institute at Medical University of Plovdiv, 4002 Plovdiv, Bulgaria; 5 Medical Simulation Training Center, Medical University of Plovdiv, 4002 Plovdiv, Bulgaria

**Keywords:** *CTBP1* mutation, HADDTS, Mito Stress Test, Seahorse, mitochondrial dysfunction, mitochondria-targeted nutritional supplementation

## Abstract

The C-terminal binding protein 1 (CTBP1) is a transcriptional corepressor with a major role in nervous system growth and development. There are only 20 published cases with *CTBP1* mutations, displaying a phenotype of Hypotonia, Ataxia, Developmental Delay and Tooth enamel defect Syndrome (HADDTS). Histochemical evidence of decreased mitochondrial respiratory chain activity has been previously reported, but comprehensive data on the metabolic phenotype assessed by various cellular respiration parameters are still missing. We present a 10-year-old female with typical HADDTS features, harboring the most reported de novo heterozygous *CTBP1* mutation c.991C>T. To elucidate her metabolic phenotype, we quantified mitochondrial respiration in peripheral blood mononuclear cells (PBMCs) utilizing an analyzer for assessing mitochondrial function (Seahorse XFp). Real-time metabolic assays revealed profound mitochondrial dysfunction with significantly attenuated maximal respiration and spare respiratory capacity compared to neurotypical controls. Following mitochondria-targeted nutritional support for one-year measurable bioenergetic improvements and reduced number of respiratory infections were registered. However, neurological recovery and new skill acquisition were not observed. We present a novel case of *CTBP1*-related neurodevelopmental disorder and demonstrate, for the first time, the application of non-invasive, real-time mitochondrial functional assessment in this setting, providing additional evidence for mitochondrial dysfunction in HADDTS.

## 1. Introduction

C-terminal binding proteins, CTBP1 and CTBP2, are two highly conserved proteins ubiquitously expressed in all human tissues. Their main role is to act as transcriptional corepressors by forming functional dimers with chromatin-modifying enzymes, DNA-binding proteins, chromodomain-containing proteins, and corepressors in repressor element silencing transcription factor (CoREST) proteins. The latter attaches to the substrate-binding domains, which contain the Pro-X-Asp-Leu-Ser (PXDLS) binding sequence—a highly specific protein-binding interface [1].

Both *CTBP1* and *CTBP2* are involved in nervous system growth and development. Homozygous mutations in the *CTBP2* gene are considered lethal and no reports with sequence variants of this gene are available in humans [1]. The *CTBP1* gene is located on chromosome 4p and is crucial for nervous system development via regulation of genes involved in neuronal growth, survival, membrane excitability, synaptic transmission and plasticity [1,2]. It also participates in the regulation of apoptosis and oncogenesis, as high *CTBP1* expression is shown to attenuate programmed cell death and enhance neuronal activity [3]. Importantly, the CTBP1 protein is involved in maintaining mitochondrial integrity and its loss may lead to mitochondrial dysfunction [4].

*CTBP1* mutations have been found to cause an extremely rare and distinctive neurodevelopmental phenotype characterized by Hypotonia, Ataxia, Developmental Delay and Tooth enamel defect Syndrome (HADDTS) [5]. Only 20 cases have been reported to date in the scientific literature [2,3,4,6,7,8,9,10,11,12]. Few cases with mitochondrial dysfunction in HADDTS have been described, diagnosed by skeletal muscle biopsy and enzyme histochemistry [2,6].

Here, we report a case of a patient with HADDTS and genetically confirmed c.991C>T de novo heterozygous *CTBP1* mutation and present a biochemical approach to evaluate mitochondrial function in real time, therefore offering novel insights into the cellular bioenergetics of *CTBP1* mutations.

## 2. Results

### 2.1. Case Presentation

#### 2.1.1. Clinical Data and Disease Course

We report a 10-year-old female of Caucasian descent. She was born as the first child of non-consanguineous parents after a full-term pregnancy and uncomplicated vaginal delivery with a birth weight of 3000 g. There was no family history of note and perinatal anamnesis was also uneventful. She was able to roll at 6 months and to sit independently at 8 months. Unaided walking with waddling and a wide-based gait was achieved at 14 months. She managed to climb stairs with support at 18 months but had difficulties rising from the floor. Running was never achieved. Language acquisition was delayed, with first words appearing at 27 months, first sentences at 36 months and asking questions at 42 months. A spontaneous smile was present since age 2 months and pointing at objects at 36 months.

Her first admission to the Department of Pediatrics was at the age of 17 months for a waddling gait and delayed expressive language development. Coarse facial features, short neck, wide rib cage, umbilical hernia and subtle hepatomegaly were noted. Muscle tone and strength were assessed as normal.

In the following years, regression in neurodevelopment was noted, accompanied by ataxia, decreased muscle tone and strength. At 5 years, ambulation was possible only with hand support and by the age of 6 years, she lost the ability to walk. Severe kyphoscoliosis developed, needing surgical correction at 8 years. At 9 years, the patient lost the ability to point towards objects, to say words, to sit independently and to roll. She has been suffering from recurrent respiratory infections throughout all these years.

Upon her rehospitalization at 9 years, she was found undernourished with a body weight of 14 kg and a body mass index far below the third centile, prompting gastrostomy tube placement. Chronic respiratory failure and several apneic episodes led to tracheostomy and intermittent ventilatory support. Kyphoscoliosis, multiple contractures and dystrophic teeth were also found. Her neurological examination was remarkable for multiple flexion contractures, generalized muscle hypotrophy with no active movements and limited passive mobility in the ankles. Generalized deep tendon hypo/areflexia, with negative Babinski and Rossolimo signs, was present. Exotropia of the left eye and hyperactive oculocephalic reflex were also noted.

#### 2.1.2. Genetic Findings

Whole exome sequencing (WES) revealed a de novo heterozygous pathogenic variant in the CTBP1 gene, c.991C>T, p.(Arg331Trp), consistent with the clinical phenotype, along with a de novo variant of unknown significance—ATP1A3 gene mutation, c.83A>C, p.(Glu28Ala).

#### 2.1.3. Neuroimaging and Paraclinical Findings

Magnetic resonance imaging conducted at the age of 6 years revealed cerebellar atrophy. Recent blood gas analysis was dominated by hypoxia (pO2 55–77 mmHg), accompanied by respiratory alkalosis or acidosis (pCO2 32–76 mmHg). Lactate elevation was infrequent and mild (2.2–6 mmol/L) and was only registered during her stays in the pediatric intensive care unit for episodes of apnea and bradycardia. Clinical and ultrasonographic exams and biochemical tests revealed no heart or liver pathology.

#### 2.1.4. Assessment of Mitochondrial Function

**Figure 1:**
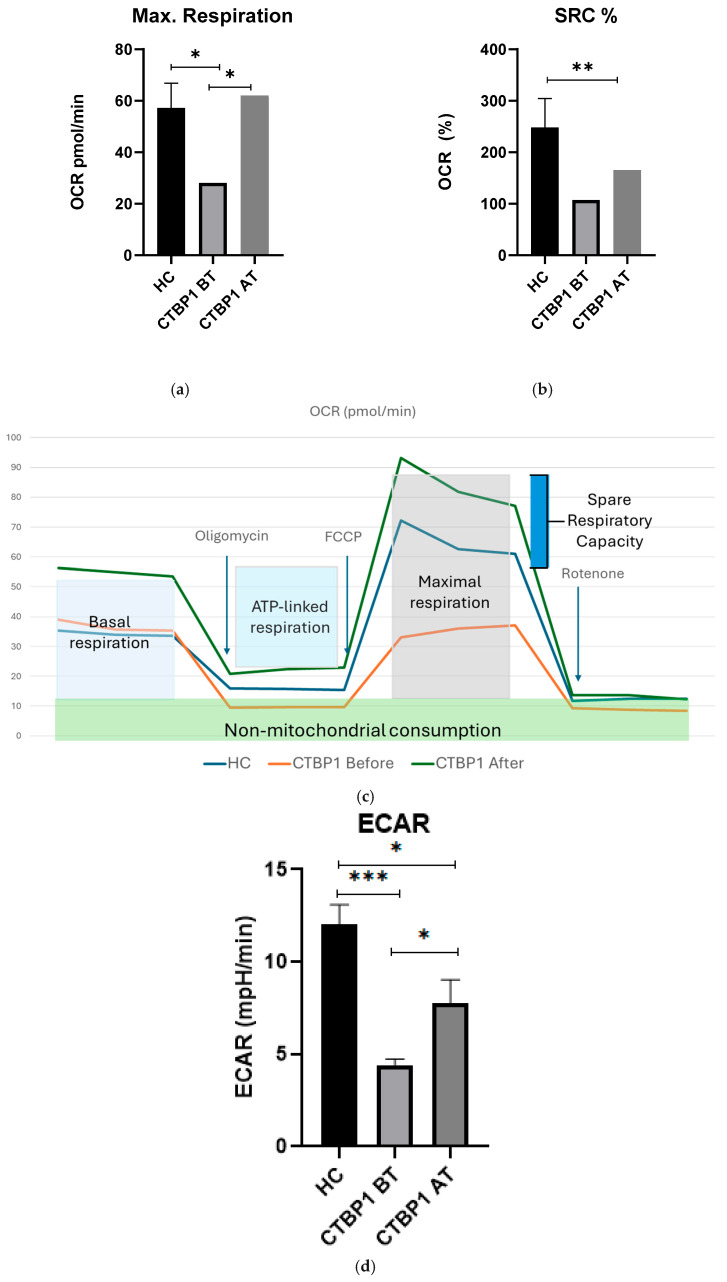
Real-time assessment of mitochondrial respiration in PBMCs from a CTBP1 patient before treatment (BT) and after treatment (AT) compared to healthy controls (HC) obtained by Seahorse XFp analysis: (**a**) Maximal respiration is significantly decreased; (**b**) Spare respiratory capacity is severely impaired; (**c**) Oxygen consumption rate (OCR) traces show reduced maximal respiration and reduced spare respiratory capacity before treatment; (**d**) Extracellular acidification rate (ECAR) demonstrates reduced glycolytic activity compared with HC with improvement after treatment. Error bars represent standard deviation (SD). * *p* < 0.05, ** *p* < 0.01, *** *p* < 0.001.

Real-time evaluation of mitochondrial function (Mito Stress Test) was performed via an analyzer for assessing cellular respiration. The initial Mito Stress Test at the age of 9 years revealed severely impaired mitochondrial function. Maximal respiration (MR) was measured at 28.77 pmol/min, significantly lower compared to 52.36 pmol/min in healthy controls (HC) (*p* = 0.02) (Figure 1a,c). Spare respiratory capacity (SRC) was reduced twofold compared to HC (CTBP1 = 107% versus HC = 261%) (Figure 1b,c). The patient exhibited an SRC% of 107%, indicating that maximal respiration exceeded basal respiration by only 7%. This suggests a critically low mitochondrial reserve, which may reflect impaired mitochondrial function in the patient’s PBMCs. Additionally, extracellular acidification rate (ECAR) was significantly decreased, indicating reduced glycolytic rate and glycolytic flux, consistent with a global metabolic dysfunction (Figure 1d). Although ECAR is commonly used as a proxy for glycolytic activity, it may be influenced by PBMC activation state. All samples were obtained under clinically stable conditions.

### 2.2. Metabolic Treatment

Based on the available mitochondrial dysfunction literature data, at 9 years, the patient was initiated on adjunctive metabolic supplementation targeting cellular bioenergetics and redox balance. The therapeutic regimen consisted of vitamin C (100 mg/day), riboflavin (vitamin B2, 50 mg/day), vitamin E (200 mg/day), and coenzyme Q10 (200 mg/day). The selection of these agents was based on their established roles in mitochondrial physiology [13,14,15]. Riboflavin is an essential cofactor for flavoprotein-dependent enzymes within the mitochondrial respiratory chain [16]. Coenzyme Q10 functions as a key electron carrier between complexes I/II and III and is critical for efficient oxidative phosphorylation [13,17]. Vitamins C and E contribute to antioxidant defense and membrane stabilization, thereby mitigating oxidative damage [15].

### 2.3. Follow-Up and Response to Treatment

One year following the initiation of the mitochondria-targeted nutritional supplementation, at the age of 10, the patient’s parents reported a reduction in the frequency of respiratory infections from once per month to twice per year. Despite this, there was no improvement in motor function and communication.

The follow-up Mito Stress Test demonstrated an improvement in mitochondrial function, with MR increasing to 62.65 pmol/min and SRC rising to 165% relative to baseline measurements (Figure 1a–c). ECAR also showed an increase to 7.76 mpH/min, suggesting enhanced glycolytic activity post-treatment (Figure 1d). Following treatment, the patient’s MR increased to 62.65 pmol/min, exceeding the basal control mean (52.36 pmol/min).

## 3. Discussion

*CTBP1* pathogenic mutations are known to cause hypotonia, ataxia, developmental delay and tooth enamel defect syndrome known as HADDTS—an extremely rare autosomal dominant disorder with only 20 cases reported to date in the scientific literature (Supplementary Table S1). HADDTS was first described by Beck et al. in 2016 in four unrelated individuals sharing a de novo c.991C>T mutation in *CTBP1* [5]. A limited number of pathogenic variants of *CTBP1* are known to cause this syndrome. The most frequent is c.991 C>T (p.Arg331Trp), but c.1024 C>T (p.Arg342Trp) and c.1315_1316delCA (p.Gln439ValfsTer84) are also associated with identical neurodevelopmental phenotype [10,11]. Novel c.371C>T *CTBP1* variant has also been reported in a child with distinctive facial features, developmental delay, but without hypotonia, ataxic gait, or tooth enamel defect [3].

Apart from its key functions in neural cell growth and development, *CTBP1* is also involved in maintaining mitochondrial integrity and its loss may lead to mitochondrial dysfunction. Supposed mechanisms act through disrupted transcription of genes crucial for cell energetics, mitochondrial function and apoptosis [4]. CTBP1 protein, although not localized in the mitochondria, acts as a metabolic sensor by differentially binding to NADH depending on the cellular energetic status and thus regulating downstream gene transcription. *CTBP1*-knockout mouse models demonstrated increased abnormal mitochondrial morphology and function and sensitivity to apoptosis in low glucose conditions [18]. CTBP1 protein also regulates the expression of proapoptotic genes such as Bcl-2-associated X protein (Bax), associating with its promoter region in glucose-rich conditions, and dissociating in case of glucose deprivation, respectively. If the CTBP1 protein is absent or damaged, Bax expression is disinhibited and cell death increases. Furthermore, fibroblasts from individuals with a pathogenic *CTBP1* mutation demonstrate higher rates of apoptosis in glucose-deficient conditions compared to HC, which corresponds to the elevated proapoptotic protein levels in the patient group [4]. All this indicates that the *CTBP1* gene is essential for cellular bioenergetics and programmed cell death by regulating gene expression in response to cellular metabolic status [18]. Notably, CTBP1 protein may also disrupt the expression of nuclear genes involved in mitochondrial functions, including those essential for mitochondrial protein synthesis machinery [6]. 

Several individual reports have already investigated mitochondrial dysfunction in HADDTS. Sommerville et al. described a 16-year-old patient with developmental regression, intellectual disability, general muscle hypotonia with elbow and wrist contractures, and cerebellar atrophy. WES revealed the most reported pathogenic c.991C>T p.(Arg331Trp) *CTBP1* mutation arising de novo in a heterozygous state. Skeletal muscle biopsy with oxidative enzyme histochemical analysis was performed, demonstrating patchy loss of COX activity and clumped SDH reactivity. Biochemical analysis of mitochondrial respiratory chain activities showed a significant reduction in complex I and IV activities, but sparing of complex II activity. The patient was initially suspected to have early-onset mitochondrial disease, but no pathogenic variants were found in the nuclear genes that encode mitochondrial proteins, nor in the mitochondrial genome. Mitochondrial DNA depletion or rearrangement was also excluded [6]. A 6-year-old female with global developmental delay, central hypotonia, cerebellar dysfunction and poor weight gain was reported by Wong et al. WES identified the common missense mutation c.991C>T p.(Arg331Trp) in the *CTBP1* gene. No pathogenic variants of other genes were found. Respiratory chain enzymological studies performed on skeletal muscle biopsy specimens displayed decreased complex I, II + III and IV activity, with the most pronounced reduction affecting complex IV. The patient was trialed on mitochondria-targeted nutritional supplementation consisting of Q10, ascorbic acid, riboflavin and vitamin E to boost respiratory chain function. After its initiation, the parents reported less frequent infections [2]. Furthermore, Kadhim et al. registered the presence of mitochondrial alterations from muscle biopsy and enzyme histochemistry in a patient with de novo *CTBP1* heterozygous mutation c.1024 C>T [11]. Increased serum lactate levels have also been described in patients with *CTBP1* mutations [2]. All these data underscore *CTBP1* mutations as a viable cause for secondary mitochondrial dysfunction.

The *ATP1A3* gene is located on chromosome 19q and encodes the alpha-3 subunit of the Na^+^/K^+^ ATPase, which is a critical membrane transporter for neuronal cells. It is essential for maintaining the electrochemical gradients of sodium and potassium across the cellular membrane and regulates neuronal excitability, neurotransmitter reuptake and cell volume [19]. The alpha-3 subunit is expressed predominantly in the central nervous system and acts as a rescue pump during periods of high neuronal activity by rapidly restoring ion gradients. Mutations in *ATP1A3* are most commonly de novo heterozygous missense mutations, but inherited cases and mosaicism have also been reported [20]. Pathogenic variants of this gene are associated with a broad spectrum of neurological disorders, most notably alternating hemiplegia of childhood (AHC), rapid-onset dystonia parkinsonism (RDP) and cerebellar ataxia, areflexia, pes cavus, optic atrophy, and sensorineural hearing loss (CAPOS) syndrome. These disorders often present with acute onset and paroxysmal episodes, often triggered by fever or stress [19]. Although we find that the *CTBP1* mutation sufficiently explains the phenotype, the disease progression and the mitochondrial dysfunction in the reported patient, the potential synergistic or modifying effect of the *ATP1A3* variant cannot be excluded, as pathogenic mutations in the *ATP1A3* gene might result in heterogeneous overlapping neurological, cognitive and neurobehavioral findings. The ATP1A3 variant (c.83A>C; p.Glu28Ala) found in our patient was evaluated using multiple in silico prediction tools, including SIFT, PolyPhen-2, and MutationTaster (Supplementary Figure S1). These consistently predict a benign effect on protein function, with no strong evidence supporting a damaging impact or relation to ataxia and neurological regression. From a biochemical perspective, the substitution involves a change from a negatively charged glutamic acid (Glu) to a small non-polar alanine (Ala), which represents a physicochemical alteration; however, the affected residue is located in the N-terminal cytoplasmic region, outside the well-characterized transmembrane domains and known functional hotspots of ATP1A3. Of note, disease-associated ATP1A3 variants are predominantly clustered in highly conserved transmembrane and catalytic regions critical for Na^+^/K^+^-ATPase activity (e.g., p.Asp801Asn, p.Glu815Lys), where mutations are known to disrupt ion transport and lead to neurological phenotypes. In contrast, p.Glu28Ala lies outside these regions and does not affect known functional or structural domains essential for pump activity. The variant is not reported in ClinVar as pathogenic, and there is no current evidence linking changes at this position to disease. Taken together with the in silico predictions and its location outside mutational hotspots, the available data argue against a major functional effect. Additionally, the link between *ATP1A3* and mitochondrial function has been poorly investigated and is limited to experimental models. The *ATP1A3* gene encodes a cellular membrane protein that is not related, at least directly, to mitochondria (https://www.ncbi.nlm.nih.gov/gene/478, accessed on 12 April 2026). Only one study assumes that pathogenic *ATP1A3* variants (p.R756C, specifically) lead to reduced heat-shock protein synthesis, decreased mitochondrial inner membrane potential and impaired mitochondrial stability solely during heat-stress [21]. However, no characterization of cellular respiration and respiratory chain enzymes in *ATP1A3* patients has been made so far. Finally, we attribute a minor or no role of the c.83A>C, p.(Glu28Ala) *ATP1A3* mutation in our patient due to the following reasons: (1) The phenotype does not correspond to any of the known phenotypes of *ATP1A3*-related disorders; (2) The *ATP1A3* variant in our patient is not pathogenic according to ClinVar and in silico analysis; (3) There are no available data on clinically significant affection of ATP1A3 protein deficiency on mitochondrial respiration.

The cited above limited evidence of mitochondrial dysfunction in patients with HADDTS has been obtained mainly by means of skeletal muscle biopsy and enzyme histochemistry. This method remains the most reliable tool to evaluate mitochondrial integrity and respiratory chain enzyme activity. However, being invasive, complex and time-consuming, it poses some serious disadvantages. Hence, novel simplified time-efficient and non-invasive methods are needed to optimize the identification of mitochondrial dysfunction.

One of the key advantages of the analyzer for assessing mitochondrial function is that it allows investigation of any type of living cell’s metabolism, including easily accessible cells such as peripheral blood mononuclear cells (PBMCs). Interestingly, PBMCs are considered suitable models in studies of neuronal cell energy metabolism due to similarities between the two cell types. Both types of cells express a lot of common receptors for cytokines, neurotrophins, and neurotransmitters, which suggest similar reactions to external stimuli. Furthermore, microglial cells, which are known to be key players in central nervous system integrity, have an intrinsic link to the immune system. This is reinforced by the fact that neuroinflammation and neurodegeneration are tightly connected to microglial activation and central nervous system infiltration with peripheral immune cells. Finally, mutations in the nuclear genome affect all cell types equally. Hence, if there is mitochondrial dysfunction due to a nuclear gene mutation, it will be present and affect both neural and immune cells [22,23].

In the reported patient, the Mito Stress Test demonstrated significantly decreased MR and SRC compared to HC, reflecting reduced activity of the mitochondrial electron transport chain and limited compensatory capacity in energy-demanding conditions (e.g., infections). Importantly, these functional data correspond with the previously reported decreased activities of mitochondrial complexes I and IV in HADDTS patients, as determined by muscle biopsy, suggesting complex regulation of mitochondrial function and a putative role of additional pathogenic mechanisms [2,6].

Our findings corroborate the emerging recognition of mitochondrial dysfunction as a key pathophysiological component in neurodevelopmental disorders with genetic etiology [24,25]. The severely decreased ECAR further highlights a diminished glycolytic flux, suggesting an overall metabolic dysfunction, characterized by reduced glycolytic flux and compromised cellular respiration.

Metabolic supplementation with a nutritional supplementation comprising vitamins C, B2, E, and coenzyme Q10 was associated with a partial restoration of mitochondrial function, reflected by significant increases in MR and SRC, alongside improved glycolytic activity. This aligns with previous evidence supporting the efficacy of targeted metabolic therapies in enhancing mitochondrial performance and redox balance in patients with mitochondrial impairments [13,26,27]. While clinical improvements in motor function, speech, cognition and social interaction were not observed, a reduction in respiratory infections was reported. Although the latter can be attributed to tracheostomy placement and ventilatory support, systemic benefits of improved cellular energetics and antioxidant capacity may also play an additional role.

Indeed, the lack of functional neurological improvement suggests that mitochondrial dysfunction might be only one facet of the complex CTBP1-related phenotype or that earlier intervention may be necessary for meaningful clinical outcomes [23,24].

## 4. Materials and Methods

### 4.1. Participant Selection

In order to assess mitochondrial function correctly, 10 healthy controls (HC) were examined in parallel with the patient. HC includes children of both sexes with a mean age of 7.6 years. Potential effects of age, sex, or other confounders on the measurements were not formally assessed. They consisted of Bulgarian children with typical neurodevelopment and no history of neurological, psychiatric, or developmental disorders. Participants were eligible for inclusion as they met the following criteria: age between 2 and 11 years; no intake of vitamins, mineral supplements, immunomodulators, antibiotics, or similar substances within three months prior to enrollment; absence of acute illness or epileptic seizures; and no evidence of chronic medical conditions, like gastrointestinal disorders, chronic infections, bronchial asthma, or diabetes mellitus. All healthy participants underwent clinical screening to confirm overall good health and typical development.

Peripheral blood samples were obtained from HC and from the child with the *CTBP1* mutation as a part of routine clinical laboratory investigations, involving no additional risk beyond standard venipuncture procedures. Samples were drawn by venipuncture into EDTA-containing Vacutainer monovettes (S-Monovette 2.6 mL, Sarstedt AG & Co. KG, Nümbrecht, Germany).

Written informed consent was obtained from the parents. Assent from the child is unapplicable due to age and mental status. This study was conducted in accordance with the Declaration of Helsinki and was approved by the Ethics Committee at the Medical University of Plovdiv (Protocol No.2/07.02.2024).

### 4.2. Sample Preparation

Peripheral blood mononuclear cells (PBMCs) were obtained within 2 h of venipuncture by density gradient centrifugation using Histopaque-1077 (Sigma-Aldrich) following the manufacturer’s recommendations. The isolated cells were maintained overnight to allow recovery from isolation stress in RPMI-1640 medium (Pan Biotech, Cat. No. P04-22100) supplemented with 10% fetal bovine serum (FBS) (Sigma-Aldrich, St. Louis, MO, USA) and 1% penicillin/streptomycin (PAN-Biotech, Aidenbach, Germany) under standard culture conditions (37 °C, 5% CO_2_, humidified atmosphere). PBMC viability was assessed using an automated cell counter (LUNA™, Logos Biosystems, Anyang, Republic of Korea) both after isolation and prior to the assay. High cell viability was consistently observed, indicating that the overnight resting period did not result in substantial loss of viable cells. All samples of healthy control (n = 10) underwent the same experimental procedure as the patient sample, including overnight incubation in RPMI-1640 under identical conditions prior to analysis. Prior to metabolic analysis, cells were plated at a density of 2 × 10^5^ viable cells per well in Seahorse XFp 8-well microplates.

### 4.3. Mito Stress Test

Mitochondrial function was assessed in freshly isolated living cells (minimizing artifacts caused by cryopreservation) using the Seahorse XFp Analyzer (Agilent Technologies, Santa Clara, CA, USA), a platform that enables rapid and sensitive measurement of cellular bioenergetics. All samples were processed under identical conditions, allowing for relative comparisons between groups.

Basal oxygen consumption rate (OCR) and extracellular acidification rate (ECAR) were measured initially. Sequential injections of mitochondrial inhibitors were then applied to assess mitochondrial respiration and glycolytic function: oligomycin (1.5 µM) to inhibit ATP synthase and determine ATP-linked respiration and proton leak; FCCP (2.0 µM) to uncouple mitochondria and induce maximal respiration; rotenone (0.5 µM) to inhibit complex I (antimycin A was not included) and measure non-mitochondrial respiration. Key bioenergetic parameters derived from OCR include basal respiration, ATP-linked respiration, proton leak, maximal respiratory capacity, spare respiratory capacity, spare respiratory capacity % and non-mitochondrial respiration (Figure 1c). Spare respiratory capacity % (SRC%) is presented as a percentage relative to basal respiration, as calculated by the Seahorse XF Analyzer software version 2.6.3.5.

ECAR was used as an indirect indicator of glycolytic activity, reflecting extracellular acidification mainly due to lactate production during anaerobic glycolysis.

The samples were analyzed in triplicate, and average values were used for statistical evaluation. Data processing and calculation of bioenergetic parameters were performed using Seahorse Wave software v2.6.3.5 (Agilent Technologies, Santa Clara, CA, USA).

### 4.4. Statistical Analysis

Raw OCR and ECAR data were initially processed using the Seahorse XF Mito Stress report generators. Bioenergetic parameters were calculated and visualized using Seahorse Wave Desktop 2.6.3.5 (Agilent Technologies). Seahorse XF data were analyzed using Wave software version 2.6.3.5 (available from Agilent Technologies: https://www.agilent.com/) to calculate key bioenergetic parameters, including basal respiration, ATP-linked respiration, proton leak, maximal respiratory capacity, spare respiratory capacity, and non-mitochondrial respiration. For each sample, measurements were performed in triplicate wells, and the mean value per biological sample was used for statistical analysis. All data are presented as mean ± standard deviation (SD). Statistical analyses were performed using appropriate statistical software, GraphPad Prism 10.2.3 (403) (1992–2024 GraphPad Software, LLC, San Diego, CA, USA). Data distribution was assessed for normality prior to analysis. Differences between the patient and the HC were evaluated using an unpaired two-tailed Student’s *t*-test for normally distributed data or the Mann–Whitney U test for non-normally distributed data. For comparisons involving more than two conditions, one-way or two-way analysis of variance followed by appropriate post hoc tests was applied. A *p*-value < 0.05 was considered statistically significant. Comparisons involving the single patient before and after therapy acknowledge the limitations of statistical inference for n = 1.

### 4.5. Limitations

Given the single-patient nature of this study, inferential statistics should be interpreted with caution. The healthy controls were selected based on strict inclusion criteria such as absence of illness and no use of supplements, probiotics, antibiotics, or other agents affecting mitochondrial function. However, the lack of detailed immune phenotyping should be considered when interpreting these findings. While PBMCs provide a useful and accessible model for assessing mitochondrial function, their metabolic profile may not reflect entirely the bioenergetic state of central nervous system cells.

## 5. Conclusions

This study expands the limited literature data for CTBP1-related neurodevelopmental disorder and provides the first real-time functional characterization of cellular bioenergetics in a patient with HADDTS using Seahorse-based mitochondrial analysis. Assessment of PBMCs revealed alterations in mitochondrial respiration consistent with prior histochemical findings, yet not detectable by conventional systemic biomarkers such as lactate. Our results support the concept that mitochondrial dysfunction may contribute to clinical severity in HADDTS. The demonstration of measurable bioenergetic improvements following metabolic supplementation without concurrent clinical improvements, however, suggests that mitochondrial dysfunction is just one component of the underlying pathogenetic mechanisms. Future clinical trials with larger cohorts and longer follow-up are warranted to delineate the extent to which metabolic interventions can modify disease trajectory and functional outcomes.

## Data Availability

The original contributions presented in this study are included in the article/Supplementary Materials. Further inquiries can be directed to the corresponding authors.

## Supplementary Materials

The following supporting information can be downloaded at: https://www.mdpi.com/article/10.3390/ijms27094003/s1.

## Abbreviations

The following abbreviations are used in this manuscript:

CTBP1	C-Terminal Binding Protein 1
HADDTS	Hypotonia, Ataxia, Developmental Delay and Tooth enamel defect Syndrome
PBMCs	Peripheral Blood Mononuclear Cells
WES	Whole Exome Sequencing
SRC	Spare Respiratory Capacity
MR	Maximal Respiration
ECAR	Extracellular Acidification Rate
HC	Healthy Controls
