# Supplementary material for: A Rare CTBP1-Related Neurodevelopmental Disorder Is Associated with Impaired Mitochondrial Bioenergetics: A Functional Case Report
Source: Int J Mol Sci. 2026 Apr 29;27(9):4003. doi: 10.3390/ijms27094003 (PMC13163389; doi:10.3390/ijms27094003)
Supplement: Supplementary file 1 [file ijms-27-04003-s001.zip › Supplementary Figure S1.pdf]

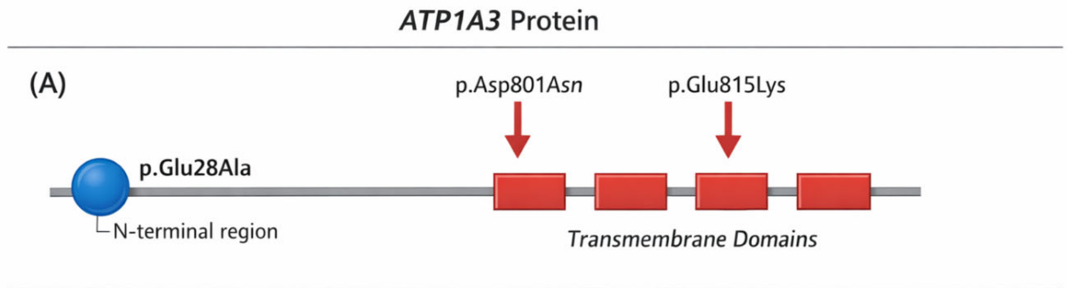

(B) *In Silico* Predictions

Tool	Prediction
SIFT	Tolerated
PolyPhen-2	**Benign**
MutationTaster	Polymorphism

Supplementary Figure S1. In silico characterization of the ATP1A3 variant (p.Glu28Ala). (**A**) Schematic representation of the ATP1A3 protein showing the location of the p.Glu28Ala variant in the N-terminal region, outside known mutational hotspots. In contrast, pathogenic variants such as p.Asp801Asn and p.Glu815Lys are located within functionally critical transmembrane domains. (**B**) In silico prediction tools (SIFT, PolyPhen-2, MutationTaster) consistently suggest a likely benign effect of the p.Glu28Ala substitution.
