# Supplementary material for: A Rare CTBP1-Related Neurodevelopmental Disorder Is Associated with Impaired Mitochondrial Bioenergetics: A Functional Case Report
Source: Int J Mol Sci. 2026 Apr 29;27(9):4003. doi: 10.3390/ijms27094003 (PMC13163389; doi:10.3390/ijms27094003)
Supplement: Supplementary file 1 [file ijms-27-04003-s001.zip › Supplementary Table S1.pdf]

Supplementary Table S1. Data of all reported HADDTS cases and the present case.

Authors / Data	Sanchez Marco et al., 2025 * [1]	Colomer et al. 2017 [2]	El Houwayek E et al. 2020 [3]	Wong et al. 2022 [4]	Present case
Number of cases	17	1	1	1	1
Age at description (years)	3-25 (Mean 11.6)	8	16	6	10
Gender	6F 11M	F	M	F	F
CTBP1 variant	15 with c.991C>T; 1 with c.1315-1316delCA; 1 with c.1024 C>T (p.Arg342Trp)	c.991C>T	c.991C>T	c.991C>T	c.991C>T
Global developmental delay	+	+	+	+	+
Speech impairment	8/14 dysarthria; 3 N/A	N/A	N/A	N/A	-
Ataxia	15/17	+	+	+	+
Hypotonia	15/17	+	+	+	+
Seizures	N/A	-	-	-	-
Dental abnormality	14/15, + enamel defects 1/15 only enamel defects	+ (enamel defects)	+ (teeth discoloration)	+ (reduced mineralization) with discoloration0	+ (dystrophic teeth)
Low weight gain	9/10	N/A	N/A	+	+
Other features	Dysmorphic features: 8/8; Contractures: 3/14; Nystagmus: 4/14 WPW syndrome: 1/14	Scoliosis	Scoliosis	Nystagmus	Dysmorphism Kyphoscoliosis Contractures Left eye exotropia
Brain MRI	Cerebellar pathology: 11/15	N/A	Cerebellar atrophy	Cerebellar atrophy	Cerebellar atrophy
ENG/EMG for myopathy	5/6	+	+	N/A	N/A
ENG/EMG for neuropathy	1/6	-	-	N/A	N/A
Myopathy on muscle biopsy	8/8	+	+	+	N/A
Immunohistochemistry of respiratory chain enzymes	Decreased complex I and IV activity 1/1	N/A	N/A	Decreased complex IV, I and II + III activity	N/A
Mitochondrial functional analysis	N/A	N/A	N/A	N/A	MR 28.77 pmol/min SRC 107% ECAR 4.41 mpH/min (After metabolic treatment: MR 62.65 pmol/min SRC 165% ECAR 7.76 mpH/min)

*Abbreviations: * Number of cases with present sign/number of all cases with available data; M—male; F—female; (+)—present sign; (-)—absent sign; N/A—non-applicable (no available data).*

References

1. Sanchez Marco, S.B.; Pardington, E.; Monaghan, M.; Spaull, R.; Fadilah, A.; Kurian, K.; Vijayakumar, K.; Smithson, S.; Majumdar, A. Hypotonia, Ataxia, Developmental Delay and Tooth Enamel Defect Syndrome (HADDTS) due to a Heterozygous de Novo Missense Variant in *CTBP1* Identified via Whole Genome Sequencing. *Case Rep. Pediatr.* **2025**, 2025, 3604592. https://doi.org/10.1155/crpe/3604592.
2. Colomer, J.; De, B.; Ortez, C.; Jou, C.; Jiménez-Mallebrera, C.; Olivé, M.; Codina, A.; Alarcón, M.; Mamiesse, A.F.; Corbera, J.; et al. C-terminal binding protein 1 (CtBP1) deficiency, mimicking congenital myopathy during infancy. *Neuromuscul. Disord.* **2017**, *27*, S186. https://doi.org/10.1016/j.nmd.2017.06.335.
3. El Houwayek, E.; Coppens, S.; Topf, A.; Duff, J.; Kaleeta, J.; Kadhim, H.; Remiche, G.; Straub, V.; Deconinck, N. HEREDITARY NEUROPATHIES & ALS. *Neuromuscul. Disord.* **2020**, *30*, S77. https://doi.org/10.1016/j.nmd.2020.08.109.
4. Wong, W.; Balasubramaniam, S.; Wong, R.S.H.; Graf, N.; Thorburn, D.R.; McFarland, R.; Troedson, C. Mitochondrial respiratory chain dysfunction in a patient with a heterozygous de novo CTBP1 variant. *JIMD Rep.* **2022**, *63*, 546–554. https://doi.org/10.1002/jmd2.12326.
